# Bronchoalveolar cytokine profiling identifies distinct endotypes in severe recurrent wheeze of infancy

**DOI:** 10.1111/pai.70449

**Published:** 2026-08-17

**Authors:** Alice Michel, Chervin Hassel, Marc Lubrano‐Lavadera, Cédric Agossah, Laure Couderc, Slobodan Culina, Bernd Jagla, Lamia Medghoul, Isabelle Michelet, Juliette Raghani, Astrid Vabret, Christophe Marguet, Hortense Petat

**Affiliations:** ^1^ Univ Rouen Normandie, Université de Caen Normandie, INSERM, Normandie Univ, DYNAMICURE UMR 1311 Rouen France; ^2^ Paediatric Department CHU Charles Nicolle Rouen France; ^3^ Paediatric Department CHU Caen Caen France; ^4^ scBiomarkers UTechS & Bioinformatics & Biostatistics Hub, Institut Pasteur, Université Paris Cité Paris France; ^5^ Bioinformatics & Biostatistics Hub Institut Pasteur, Université Paris Cité Paris France; ^6^ Department of Virology Université de Caen Normandie, Univ Rouen Normandie, INSERM, Normandie Univ, DYNAMICURE UMR 1311, CHU Caen Caen France; ^7^ Department of Pediatrics and Adolescent Medicine, Pulmonology, Allergy and CF UnivRouen Normandie, UMR 1311, DYNAMICURE, FHU Respire, CHU Rouen Rouen France

**Keywords:** bronchoalveolar lavage, cytokines, preschool wheeze

To the Editor,

Preschool wheeze is associated with high morbidity and frequent hospitalisations despite early therapeutic intervention.^1^ It is now considered a major risk factor for the subsequent development of chronic obstructive airway disease through persistent airway inflammation and airflow limitation.^2^

However, the pathophysiology of preschool wheeze remains incompletely understood. A prevailing hypothesis considers preschool wheeze to be primarily a post‐viral respiratory disorder,^3^ typically associated with neutrophilic airway inflammation and the absence of eosinophils in bronchoalveolar lavage fluid (BALF).^4^ The recognized weak effect of inhaled steroids on this neutrophilic bronchial inflammation may contribute to poor control and a vicious cycle involving recurrent viral respiratory infections and impaired innate pulmonary immunity. Recent studies have extended the pro‐inflammatory cytokine profiles in the sputum of preschool wheezers classified by severity of the disease. The results suggest that the more symptomatic the disease, the more pronounced the INF‐γ deficiency in sputum during acute wheezing.^5^ Regarding the various pathways involved in lung inflammation, we hereby aimed to characterize the bronchoalveolar lavage cytokine profile of preschool children with severe recurrent wheeze at a steady state. Bronchoalveolar lavage was performed as part of routine clinical care during the diagnostic evaluation of preschool children aged <2 years, with severe recurrent wheeze enrolled in the prospective Microbiosthma cohort (approval number ID‐RCB: 2023‐A00777‐38), as design and protocol previously published.^6^ Severe recurrent wheeze was defined as recurrent wheezing episodes requiring specialized evaluation because of persistent respiratory symptoms despite appropriate therapy and/or recurrent severe exacerbations. Parental consent was obtained for the patients. For this pilot study, we included the first 40 children. For research purposes, 2 mL of recovered BALF were immediately transferred into 6 mL of DNA/RNA Shield (Zymo‐Research, Irvine, CA, USA), according to the manufacturer‐recommended 1:3 ratio for liquid samples, then aliquoted and stored at −80°C until analysis. Before cytokine profiling, total protein concentration was measured using the Qubit Protein Assay Kit (Invitrogen, Carlsbad, USA) and BALF samples were diluted 1:10 to meet the protein concentration range required for Olink analysis (0.5–1 mg/mL).

Cytokine profiling was performed using the Olink Target 48 Cytokines Panel (Olink, Uppsala, Sweden) according to the manufacturer's instructions. A broad panel of soluble mediators was quantified in bronchoalveolar lavage fluid comprising epithelial alarmins (TSLP, IL‐33), type 2 inflammatory mediators (IL‐13, CCL11), innate pro‐inflammatory cytokines (TNF‐α, IL‐1β, IL‐6, CXCL8), myeloid growth factors (CSF1, CSF2, CSF3), interferon‐related mediators (IFN‐γ, IL‐27, CXCL9, CXCL10, CXCL11), leukocyte chemoattractants (CCL2, CCL3, CCL4, CCL8, CXCL12), tissue remodeling factors (EGF, VEGF, HGF), and additional inflammatory regulatory mediators involved in cellular stress and apoptosis (TNFSF10 and OLR1). Olink‐generated cytokine concentrations and NPX values underwent the manufacturer's internal quality control and normalization workflow. However, no additional correction for BALF dilution was applied. Bioinformatics analyses were conducted using the Qlucore Omics Explorer software (Qlucore AB, Lund, Sweden). Statistical analyses were conducted with RStudio (R version 4.5.1). Given the exploratory nature of the study and limited sample size, analyses were primarily descriptive and unsupervised clustering was used as a hypothesis‐generating approach to explore cytokine profile heterogeneity.

Clinical and demographic characteristics are summarized in Table 1. Consistent with the inclusion criteria, the study population was relatively homogeneous. The median age was 17.4 months; 67.5% (*n* = 27) had atopic dermatitis, and 70.0% (*n* = 28) had a family history of asthma; 72.5% (*n* = 29) required at least one hospitalization. The median number of wheezing episodes during the preceding 12 months was 5, with a median of 3 courses of oral corticosteroids. All patients were daily given inhaled corticosteroid therapy, of whom 82.5% were by nebulization. In addition, 17.5% were given azithromycin three times weekly at immunomodulatory doses.

**TABLE 1:** Baseline clinical characteristics of infants with severe preschool wheezing included in the cohort.

	(*n* = 40)
Age (months)	17.5 [12, 24.75]
Birth	
Active smoking by the mother during pregnancy	10 (25%)
Gestational age	39 [38, 40]
Mode of delivery: C‐section	6 (15%)
History of atopic disease	
Age of onset of first symptoms (months)	3 [1, 4]
History of hospitalization	29 (72.5%)
Number of wheezing episodes in the last 12 months	5 [3, 10]
Number of oral corticosteroid treatments over the last 12 months	3 [2, 4.75]
Food allergies	1 (2.5%)
Atopic dermatitis	27 (67.5%)
Environment	
Child care	
Parents	17 (42.5%)
Nursery	16 (40%)
Nursemaid	7 (17.5%)
Living environment	
Rural	19 (47.5%)
Urban	13 (32.5%)
Semi‐urban	8 (20%)
Mold in the house	6 (15%)
Smokers at home	16 (40%)
Family history (father, mother, siblings)	
Asthma	28 (70%)
Food allergy	9 (22.5%)
Atopic dermatitis	27 (67.5%)
Allergic rhinitis	24 (60%)
Current symptoms at the inclusion	
Current respiratory symptoms	27 (67.5%)
Nocturnal cough	19 (47.5%)
Diurnal cough	17 (42.5%)
Exercise‐induced cough	16 (40%)
Rhinitis	8 (20%)
Shortness of breath	9 (22.5%)
Disease not controlled	15 (37.5%)
Weight break	9 (22.5%)
Treatments	
Inhaled corticosteroids	40 (100%)
Nebulisations of inhaled corticosteroids	33 (82.5%)
Montelukast	4 (10%)
Long‐term azithromycin	7 (17.5%)

BALF analysis revealed various levels of several soluble mediators. The highest concentrations were observed for CXCL8, CSF3, CXCL12, and OLR1. High concentration levels were also detected for IL‐33, CCL2, CCL3, CCL4, CXCL9, CXCL10, CXCL11, VEGFA, HGF, and TNFSF10 (Figure 1). Conversely, the lowest were INF‐γ levels.

**FIGURE 1:**
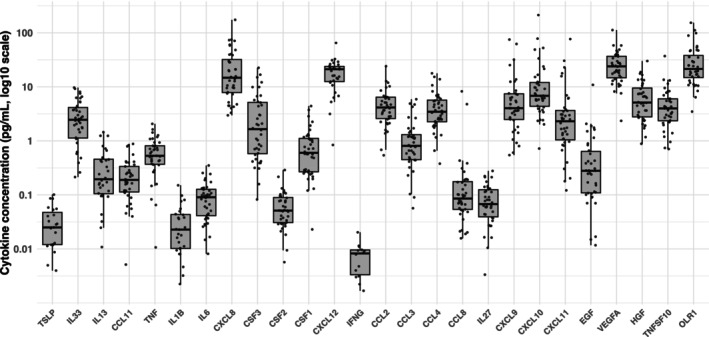
Distribution of cytokine concentrations in bronchoalveolar lavage samples. Cytokine concentrations (pg/mL) measured in bronchoalveolar lavage samples are shown as boxplots with individual data points overlaid. Data are displayed on a logarithmic scale (log10). All measurements displayed were passing quality control.

Exploratory unsupervised hierarchical clustering of z‐scored NPX values suggested three cytokine‐based clusters (Figure 2). Cluster 1 (10%) was characterized by elevated concentration levels of all measured soluble components, consistent with a broad inflammatory activation of all analyzed pathways. Cluster 2 (50%) displayed a comparable cytokine pattern with overall lower concentration levels. This suggests shared inflammatory mechanisms with Cluster 1 but of reduced intensity. In contrast, Cluster 3 (40%) exhibited a distinct inflammatory signature from the two others with comparatively lower expression of markers associated with cellular recruitment and apoptosis, and relatively higher levels of mediators suggestive of type 2 inflammation.

**FIGURE 2:**
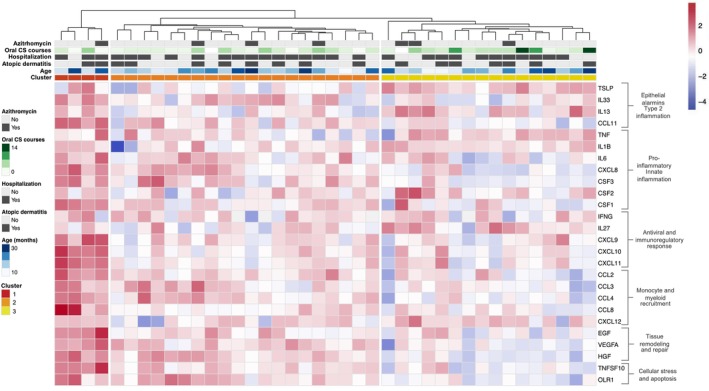
Unsupervised clustering of BALF cytokine profiles in patients with clinical characteristics. Heatmap displaying normalized protein expression (NPX) values measured using the Olink Target 48 Cytokine panel. Only assay measurements passing quality control were included. Selected cytokines of interest were analyzed after removal of analytes with no variance across samples. NPX values were standardized per cytokine using row‐wise *z*‐score transformation. Hierarchical clustering was performed using Euclidean distance and complete linkage for patients (columns), whereas cytokines (rows) were displayed according to functional categories. Red indicates relatively higher and blue relatively lower expression compared to the cohort mean. Top annotations indicate cytokine‐based cluster assignment and clinical characteristics, including age, atopic dermatitis, hospitalization history, number of oral corticosteroid courses and azithromycin treatment.

The present study describes the bronchoalveolar inflammatory profile of infants with severe recurrent wheeze at a steady state. We identified a broad innate immune signature combining epithelial alarmin activation, neutrophilic inflammation, monocyte recruitment, antiviral chemokine induction, and early tissue remodeling signals.

A central finding is the high concentration in some patients of IL‐33, an epithelial alarmin released following airway injury and viral exposure. IL‐33 is a key upstream mediator of airway inflammation and has been implicated in early‐life respiratory disease and asthma development.^7^ Its presence in BALF supports early epithelial activation in severe wheezing infants.

We observed a strong neutrophilic signature, reflected by increased CXCL8 and CSF3. This pattern is consistent with previous reports showing that severe preschool wheeze is frequently associated with neutrophil‐predominant inflammation and reduced corticosteroid responsiveness.^8^ These findings reinforce the concept that early‐life wheezing is not uniformly type 2‐driven but often dominated by innate immune pathways.

In parallel, the marked upregulation of interferon‐inducible chemokines (CXCL9, CXCL10, CXCL11) suggests sustained antiviral immune activation. These mediators are induced by IFN‐γ and are classically associated with viral respiratory infections, particularly rhinovirus, a major trigger of wheezing in early childhood and a risk factor for later asthma development.

High levels of CCL2, CCL3, CCL4, and CXCL12 indicate enhanced recruitment of monocytes and myeloid cells, potentially contributing to chronic airway inflammation and early structural changes. Longitudinal studies have linked early‐life wheezing with subsequent airway remodeling and persistent respiratory morbidity.

We further observed increased HGF levels, suggesting activation of epithelial repair pathways consistent with repeated injury–repair cycles in the developing lung. TNFSF10 high levels were therefore consistent with apoptosis‐related regulatory mechanisms involved in epithelial turnover and immune homeostasis. Increased OLR1 levels suggest a strong activation of oxidative stress–related pathways, linking airway inflammation to tissue injury responses.^9^

Hierarchical clustering further supported the hypothesis of biological heterogeneity of severe recurrent wheeze in infancy. Cluster 1 and 2 displayed a broad activation of all pathways and a severe neutrophil and other cell related inflammation and injury of epithelium. The comparison of both may represent a continuum of the same underlying inflammatory process. Cluster 3 showed a comparatively distinct cytokine profile, characterized by lower neutrophil‐related inflammation, less evidence of epithelial injury, and low expression of canonical type 2 (T2) inflammatory markers (including IL‐4, IL‐5, and IL‐13), consistent with a T2‐low inflammatory signature.

Our clustering analysis should be interpreted with caution. Although unsupervised clustering identified three distinct cytokine profiles, the relatively small sample size, particularly the limited number of patients in Cluster 1, restricts the robustness and generalizability of these findings. Therefore, these clusters should be regarded as exploratory and hypothesis‐generating rather than definitive inflammatory endotypes. Validation in larger, independent pediatric cohorts will be necessary to confirm the reproducibility and clinical relevance of these cytokine profiles.

Overall, these findings suggest that most of the severe recurrent wheeze in infancy is characterized by persistent innate immune activation with predominant antiviral and neutrophilic pathways and early evidence of airway remodeling.

Another important consideration is the potential influence of ongoing treatment on bronchoalveolar lavage cytokine profiles. All children were receiving inhaled corticosteroids, and some were also treated with long‐term azithromycin at the time of bronchoscopy. Both therapies are known to modulate airway inflammation and may therefore have influenced cytokine concentrations. Because bronchoscopy was performed in children with persistent severe recurrent wheeze despite treatment, the inflammatory profiles identified in this study are likely to represent residual treatment‐resistant inflammation superimposed on the underlying disease biology.

These results highlight that in a homogeneous population by their clinical features, different underlying biological mechanisms may arise. This would partly explain the various outcomes in severe preschool wheezers despite being given high dosing of inhaled steroids.^10^ However, in addition to the small sample size, the absence of a control group remains an important limitation, as the invasive nature of BALF collection precludes sampling from healthy infants for research purposes. Our findings should therefore be interpreted as exploratory and hypothesis‐generating rather than as definitive disease‐specific endotypes. Validation in larger cohorts with appropriate comparator groups will be required whenever feasible.

## AUTHOR CONTRIBUTIONS

**Laure Couderc:** Investigation; writing – review and editing. **Alice Michel:** Conceptualization; investigation; methodology; data curation; formal analysis; writing – original draft. **Slobodan Culina:** Formal analysis; investigation; data curation; writing – review and editing. **Bernd Jagla:** Investigation; writing – review and editing; formal analysis; data curation. **Cédric Agossah:** Investigation; writing – review and editing. **Hortense Petat:** Conceptualization; investigation; funding acquisition; writing – original draft; visualization; writing – review and editing; supervision. **Juliette Raghani:** Investigation; writing – review and editing. **Lamia Medghoul:** Investigation; writing – review and editing. **Marc Lubrano‐Lavadera:** Investigation; writing – review and editing. **Astrid Vabret:** Investigation; writing – review and editing. **Christophe Marguet:** Conceptualization; funding acquisition; methodology; visualization; writing – review and editing; supervision. **Chervin Hassel:** Conceptualization; methodology; writing – review and editing. **Isabelle Michelet:** Investigation; writing – review and editing.

## FUNDING INFORMATION

This research did not receive any specific grant from funding agencies in the public, commercial, or not‐for‐profit sectors.

## CONFLICT OF INTEREST STATEMENT

The authors declare no conflict of interest.

## Data Availability

The data that support the findings of this study are available from the corresponding author upon reasonable request.
